# A Semantic Problem Solving Environment for Integrative Parasite Research: Identification of Intervention Targets for *Trypanosoma cruzi*

**DOI:** 10.1371/journal.pntd.0001458

**Published:** 2012-01-17

**Authors:** Priti P. Parikh, Todd A. Minning, Vinh Nguyen, Sarasi Lalithsena, Amir H. Asiaee, Satya S. Sahoo, Prashant Doshi, Rick Tarleton, Amit P. Sheth

**Affiliations:** 1 The Kno.e.sis Center, Department of Computer Science and Engineering, Wright State University, Dayton, Ohio, United States of America; 2 Center for Tropical and Emerging Global Diseases, University of Georgia, Athens, Georgia, United States of America; 3 THINC Lab, Department of Computer Science, University of Georgia, Athens, Georgia, United States of America; 4 Division of Medical Informatics, School of Medicine, Case Western Reserve University, Cleveland, Ohio, United States of America; University of Melbourne, Australia

## Abstract

**Background:**

Research on the biology of parasites requires a sophisticated and integrated computational platform to query and analyze large volumes of data, representing both unpublished (internal) and public (external) data sources. Effective analysis of an integrated data resource using knowledge discovery tools would significantly aid biologists in conducting their research, for example, through identifying various intervention targets in parasites and in deciding the future direction of ongoing as well as planned projects. A key challenge in achieving this objective is the heterogeneity between the internal lab data, usually stored as flat files, Excel spreadsheets or custom-built databases, and the external databases. Reconciling the different forms of heterogeneity and effectively integrating data from disparate sources is a nontrivial task for biologists and requires a dedicated informatics infrastructure. Thus, we developed an integrated environment using Semantic Web technologies that may provide biologists the tools for managing and analyzing their data, without the need for acquiring in-depth computer science knowledge.

**Methodology/Principal Findings:**

We developed a semantic problem-solving environment (SPSE) that uses ontologies to integrate internal lab data with external resources in a Parasite Knowledge Base (PKB), which has the ability to query across these resources in a unified manner. The SPSE includes Web Ontology Language (OWL)-based ontologies, experimental data with its provenance information represented using the Resource Description Format (RDF), and a visual querying tool, Cuebee, that features integrated use of Web services. We demonstrate the use and benefit of SPSE using example queries for identifying gene knockout targets of *Trypanosoma cruzi* for vaccine development. Answers to these queries involve looking up multiple sources of data, linking them together and presenting the results.

**Conclusion/Significance:**

The SPSE facilitates parasitologists in leveraging the growing, but disparate, parasite data resources by offering an integrative platform that utilizes Semantic Web techniques, while keeping their workload increase minimal.

## Author Summary

Effective research in parasite biology requires analyzing experimental lab data in the context of constantly expanding public data resources. Integrating lab data with public resources is particularly difficult for biologists who may not possess significant computational skills to acquire and process heterogeneous data stored at different locations. Therefore, we develop a semantic problem solving environment (SPSE) that allows parasitologists to query their lab data integrated with public resources using ontologies. An ontology specifies a common vocabulary and formal relationships among the terms that describe an organism, and experimental data and processes in this case. SPSE supports capturing and querying provenance information, which is metadata on the experimental processes and data recorded for reproducibility, and includes a visual query-processing tool to formulate complex queries without learning the query language syntax. We demonstrate the significance of SPSE in identifying gene knockout targets for *T. cruzi*. The overall goal of SPSE is to help researchers discover new or existing knowledge that is implicitly present in the data but not always easily detected. Results demonstrate improved usefulness of SPSE over existing lab systems and approaches, and support for complex query design that is otherwise difficult to achieve without the knowledge of query language syntax.

## Introduction

Vast quantities of “-omics” data (proteomic, genomic, transcriptomic, metabolomic, etc.) have been created and more is being generated at an increasingly rapid pace. These data reside in internal lab-specific repositories and in a growing number of external databases such as GeneDB [1], the EupathDB databases [2] TrypanoCyc [3], and TcSNP [4] for the parasite *T. cruzi*. The abundance of data and the heterogeneous format of the internal experimental and external databases make consolidating and analyzing the data extremely challenging. As an example, to identify genes whose deletion by insertional knockout might result in avirulent and nonpathogenic (i.e. potential “vaccine” strains) of pathogenic organisms, investigators may need to integrate their internal lab specific gene expression or protein localization data with publicly available gene information sources, such as the gene ontology (GO) [5], pathway information sources such as KEGG [6], and orthologous genes sources such as TriTrypDB. Such gene, pathway, and ortholog resources are now publicly available and are often cross-referenced to each other, but are not easily integrated with “new” data being generated in various laboratories and which is not yet in these data repositories. The effective management and querying of biomedical data for knowledge discovery requires a number of specific tools and features, including: *(i)* a commonly agreed upon vocabulary of terms and relationships between the terms that facilitates data interoperability (i.e. an ontology); *(ii)* robust tools for data analysis that emphasize and utilize relationships among the data, exhibit visual interfaces and do not require advanced computer technology knowledge for their use; and *(iii)* a capability of collecting and using the provenance of data [7] for assessing its quality. A platform that additionally allows investigators to integrate and query their *internal* (unpublished) data along with external sources could expedite knowledge discovery.

EuPathDB [2] provides a prominent platform to access and query genome-scale datasets for trypanosomes and several other pathogens and is extensively used by many biologists. However, unpublished or unreleased data are generally missing from EuPathDB, and even release-ready data are not always immediately accessible, despite the frequent updating of these pathogen databases. The sensitivity and/or preliminary nature of some research findings, or other restrictions on data release, may also mean that they are not and will not in the near future be accessible through these databases. Thus, although these public resources are highly useful, they are insufficient for fast-moving research efforts.

Semantic Web approaches utilize meaningful annotations or metadata provided by the ontologies to integrate various data resources. The annotated data is often stored in a Semantic Web format called the Resource Description Format (RDF) that organizes pieces of data into subject-predicate-object triples. Semantic Web approaches have been successfully implemented in biomedical and translational research [8], [9], [10], [11], [12]. Motivated by these efforts, we created a Semantic Problem Solving Environment (SPSE) for *T. cruzi* and other related kinetoplastids. The SPSE uses biomedical ontologies as the core knowledge resource and data model along with related Semantic Web technologies. Early in this project we realized the importance of provenance to scientists' data analysis needs. Hence, domain-specific (i.e., semantic) provenance [13] has been tightly integrated within the SPSE. The SPSE provides an environment for researchers to query the data as well as the associated provenance information, all stored in a RDF triples, with or without external databases, using a visual query tool.

The SPSE is comprised of the following main components:

1. Ontologies: Two ontologies, namely the Parasite Experiment Ontology (PEO) and Ontology for Parasite Lifecycle (OPL) were developed (released through NCBO). These ontologies are integrated with existing ontologies that model related domains, such as GO and pathway ontology (PW) [14];
2. Parasite Knowledge Base (PKB): Internal experimental data from transcriptome and proteome analyses and workflow data for complex processes, such as high-throughput gene knockout studies, are semantically integrated with external databases such as KEGG and TriTrypDB and represented in RDF;
3. Semantic query processing tool, Cuebee: Cuebee is a system for querying biological data semantically. Using Cuebee, biologists may run complex queries without having to know complex query syntax. These queries may involve database access as well as Web services; and
4. A provenance management tool: Ontology-driven Web forms collect the provenance information associated with each experiment and store it in RDF, integrated with the experimental data in PKB.

A key contribution of the SPSE is that it facilitates parasitologists to execute complex queries that require internal or private data – including provenance information – and external databases in order to generate new knowledge with a minimal computing burden. This article discusses the motivation behind various components of the SPSE and how they are deployed in order to identify intervention targets in *T. cruzi*, the agent of human Chagas disease.

## Materials and Methods

**Figure 1:**
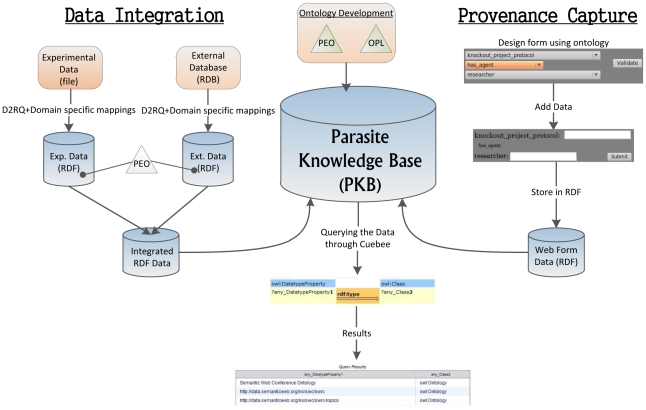
SPSE architecture showing how the various components work together.

SPSE uses two domain ontologies, PEO and OPL, to integrate data from different relational databases and incorporate provenance in the integrated dataset. The results of this provenance-enriched integrated data are then used to create a knowledge base, which can be queried using a graphical tool, Cubee. Figure 1 shows the architecture of the SPSE and the interactions between the different components. Below we describe each component of SPSE in detail and how they are being used to address research questions.

### Ontologies

An ontology is a standardized and controlled vocabulary that describes the domain, parasite lifecycle stages and experimental data in this case. We developed two ontologies, Parasite Experiment Ontology (PEO) [15] and Ontology for Parasite Lifecycle (OPL), which serve as reference schema for the non-public (lab-based) experimental data on *T. cruzi* and other related kinetoplastids. These ontologies also facilitate biological queries in the SPSE. OPL describes the parasite life-cycle stages of *T. cruzi*, *T. brucei*, and *Leishmania major*, including the host, parasitic and vector organisms, and anatomical location corresponding to each lifecycle stage. The PEO models the experimentation processes used to generate the data, the description of raw materials used, and the instruments and parameter values that influence the generation or processing of data [15]. The current version of PEO (v. 1.0) includes experimental details on the gene knockout (GKO) constructs, the gene knockout strain created, and microarray [16] and proteome [17] data generated by our studies in *T. cruzi*. In order to capture the provenance information, PEO utilized Provenir Ontology [15] as an upper level ontology. PEO reuses the classes and relationships from OPL and additionally ensures interoperability with existing biomedical ontologies published at NCBO by reusing relevant classes and properties from Sequence ontology (SO) [18] and the National Cancer Institute (NCI) thesaurus (http://ncit.nci.nih.gov) among others.

**Figure 2:**
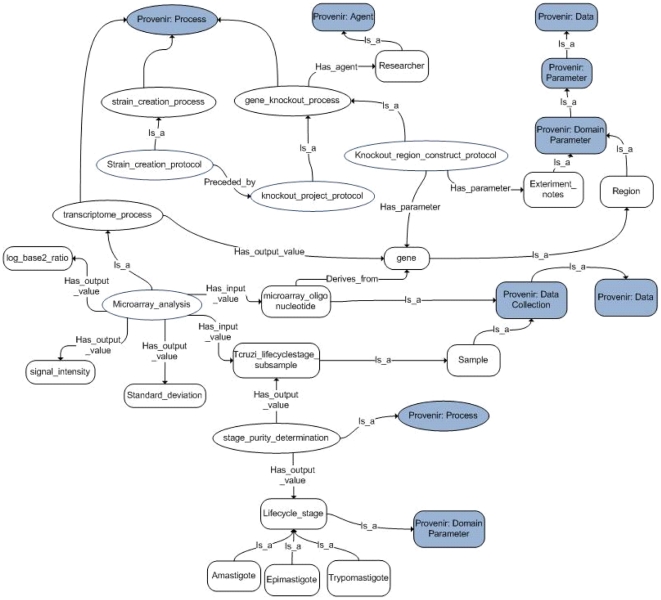
Submodules of our PEO ontology focusing on gene knockout, strain creation and microarray experiments.

Examples of other ontologies that capture experimental details are the Ontology for Biomedical Investigations (OBI) [19] and the Experimental Factor Ontology (EFO) [20]. OBI is a reference ontology for biomedical or clinical investigations and EFO is an application ontology that models the experimental factors in ArrayExpress. Recently, Cross et al. [21] identified mappings between concepts in PEO and OBI that are similar. Since EFO re-uses (and extends) many concepts from OBI, PEO concepts can be mapped to appropriate EFO concepts as well. PEO is formulated in OWL-DL [22] and contains 142 classes and 38 properties (20 object and 18 data-type properties) with a description logic (DL) expressivity of ALCHQ(D). Both PEO and OPL ontologies have been released for public use through NCBO, and are being extended with the help of researchers in parasitology. Figure 2 shows the partial schema of PEO containing some details of GKO, strain creation and microarray experiments.

### Parasite Knowledge Base

The Parasite Knowledge Base (PKB) stores non-public experimental data sets, provenance information, and external publicly available data sets in RDF with support for inferring new knowledge from the integrated data sets (Figure 1). The RDF data are stored in a system called OpenLink Virtuoso 6 (http://virtuoso.openlinksw.com/dataspace/dav/wiki/Main/), which is an open source and widely used large-scale RDF data storage system and provides an endpoint for querying the data using the Simple Protocol and RDF Query Language (SPARQL) – a Semantic Web standard for querying RDF data (http://www.w3.org/TR/rdf-sparql-query/). Below we describe each component of PKB in more detail.

#### Data Sources

Table 1 lists the data sets in the PKB along with a description of their sources. Like many other parasites, *T. cruzi* has a complicated life cycle involving insect vectors, human hosts, and mammalian reservoir hosts. Thus, one important criterion for selecting target genes for drug and vaccine studies is when and where during the parasite's life cycle a potential target gene is expressed. Genome-wide microarray [23] and proteomic [17] analyses have been performed to determine gene expression levels during all of the life-cycle stages of *T. cruzi*, and these and other data have been used to select targets for drug and vaccine studies, including the systematic creation of attenuated *T. cruzi* strains by targeted gene knockout, as described in Xu et al. [24]. Thus, the gene knockout (GKO) and strain creation datasets are comprised of the tracking data required to monitor the complex workflow of generating genetically attenuated *T. cruzi* strains.

**Table 1:** Data sources used in PKB.

Dataset	Original Source	Internal/External	Description
Gene knock out (GKO)	Tarleton Research Group	Internal	Workflow data associated with the DNA cloning steps required to generate gene knockout plasmid cassettes
Strain creation	Tarleton Research Group	Internal	Workflow data associated with the creation of gene knockout strains in *T. cruzi* by transfection of parasites with gene knockout plasmid cassettes
Microarray	Tarleton Research Group	Internal	Whole-genome relative transcript abundances for the four life-cycle stages of *T. cruzi*
Proteome	Tarleton Research Group	Internal	Protein identifications based on peptide spectra obtained from each life-cycle stage of *T. cruzi*
Ortholog	TriTrypDB and KEGG	External	Orthologous genes in organisms that are related to *T. cruzi*
Predicted signal peptide information	TriTrypDB	External	Sequence-based predictions for each *T. cruzi* annotated gene regarding the likelihood of the gene product containing a signal peptide
Transmembrane domain count information	TriTrypDB	External	Sequence-based predictions for each *T. cruzi* annotated gene regarding the presence and number, if any, of trans-membrane domains that the gene product contains
Pathway	KEGG	External	Annotations for each *T. cruzi* gene regarding their presence or not in a KEGG-annotated metabolic pathway

The heterogeneous non-public experimental data (in relational database or Excel file format) were integrated with other publicly available genome databases, such as KEGG [6] and TriTrypDB [25] as described in the next subsection. We implemented a Web client service to crawl all information related to the list of genes obtained from the experimental data sets and to query all information related to the *T. cruzi* genes from KEGG and TriTrypDB. Specifically, we obtained the associated pathway and orthologous genes from KEGG, GO annotation, and predictions of transmembrane and signal peptides from TriTrypDB (Table 1).

#### Data Integration

The construction and execution of complex biological queries over multiple data sources requires a semantically integrated data set. Specifically, all data items are annotated using concepts from the ontologies, and identical data items in two different data sets are mapped as being the same. The semantics of each data resource is implicitly associated with its metadata, which can be column names in a relational database or in a data file of tabular format. The primary challenge to integration is the heterogeneity in the data sources described in Table 1, and the lack of explicit semantics for each data source.

To address the challenge of heterogeneity of data sources, we used the approach in Sahoo et al. [11] of creating a global schema to cover all data sources and mapping the different data sources to the global schema. Data from different sources was transformed to a common RDF format that conformed to the global (PEO) schema. Since entities from external sources such as TriTrypDB and KEGG are annotated by Gene Ontology (GO) [5] and Pathway Ontology (PW) [14], we reuse concepts defined in GO and PW in the global schema. Top-level concepts from GO and PW were mapped to a class in PEO. For example, a *pathway* class was created in PEO, and then five of the top-most classes of PW—*classic_metabolic_pathway*, *disease_pathway*, *drug_pathway*, *regulatory_pathway* and *signaling_pathway*—were imported as subclasses of the *pathway* concept. This approach enables users to compose queries in terms of the global schema, while the query results include data from different sources.

While the global schema including three ontologies (PEO, GO, PW) and their mappings are comprehensive, annotations for external data sources such as transmembrane domain count, orthologous and signal peptide count is not included in the global schema yet. Therefore, for each external data entity that is not described by a respective concept in our global schema, we create a new concept or new data property in PEO. For instance, we model the transmembrane domain count as a data property of the *gene* concept.

After expanding PEO to a global schema, which describes all data sources, we used it to convert the data sources into a single, RDF-based data. Each data item becomes an RDF instance of a concept in PEO that models the data. We used Jena [26], a popular framework for building Semantic Web applications in Java, to programmatically convert all data sources into RDF. To maintain the integrity of the datasets, both PEO schema and RDF datasets were validated at the instance level automatically using the ontology reasoner called Pellet [27]. For example, Pellet validated the properties by seeking possible inconsistencies in the domain and range of each property usage.

### Answering Complex Questions Using Cuebee

Cuebee is a knowledge-driven query formulation system which facilitates the process of SPARQL-based querying of the parasite knowledge base. Although SPARQL defines a standard query language and data access protocol for use with the RDF data model, formulating queries in SPARQL is not intuitive without an intimate knowledge of the language (see example SPARQL queries in the Results and Evaluation section).

#### Cuebee: System and Functionality

**Figure 3:**
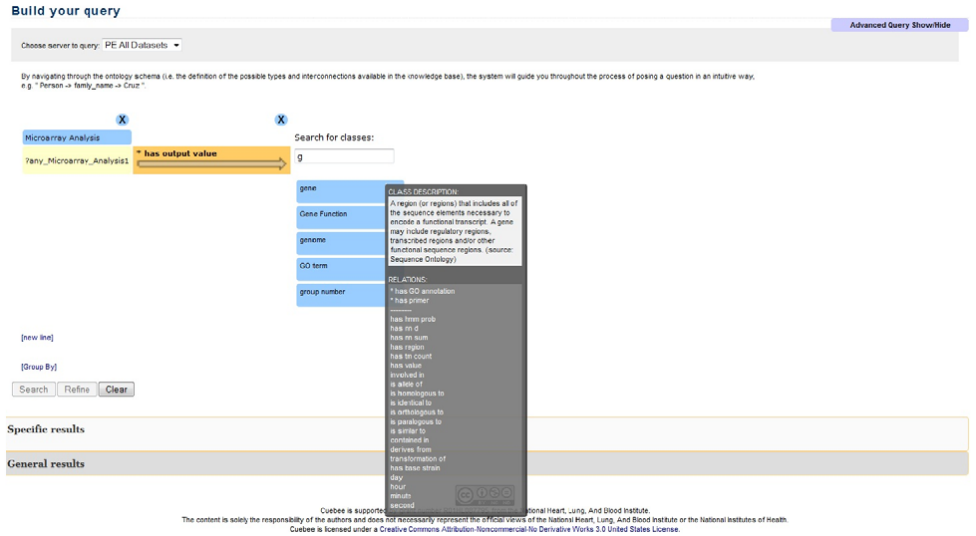
Screenshot of the Cuebee interface for query formulation. The row contains a triple (subject-predicate-object) that is required to formulate the query. The expressions over arrows represent relationships (predicates) that link the subject and object. The query formulation is initiated by first selecting the server (PE All Datasets). If the users know which particular datasets will be used for the query, they can select dataset there, such as microarray dataset, gene knockout dataset, etc. However, if the users are not sure about this, then they can select PE All Datasets, and Cuebee will try to find answers using all the datasets in Parasite Knowledge Base (PKB). Users then begin to type in the search field and Cuebee provides suggestions matching the first letters typed in a drop-down list. In this case “Microarray Analysis” is selected. The users can select specific instance of Microarray Analysis if known. Else, users can select “any_Microarray_analysis”. This will let Cuebee find answers using all the microarray data. Cuebee provides definitions on each concept (under Class Description) and more information about relationships (under Relations) as shown for the concept “gene” in this figure. Relationships that have asterix in front means that they are directly associated with the concept “gene” where “gene” acts as a subject of the triple. This information comes from the ontology, PEO in this case. Once the desired query is formulated, the users can click on Search and Cuebee will provide results under Specific results or General results section. Users can also query on the results of their first query using Refine button. The video demo on querying using Cuebee is available at: http://wiki.knoesis.org/index.php/Manuscript_Details.

Cuebee [28], available for download from http://Cuebee.sourceforge.net, allows querying of RDF data that could either be housed in conventional database management systems but published in RDF using attribute-to-concept mappings [29], or directly available in the RDF model. As we mentioned previously, the RDF data is accessible using query endpoints, allowing new data sources to be added with minimal developmental effort. Formulating queries within Cuebee utilizes an ontology schema to guide a user through the process of transforming a question into a query in a logical way. These queries are formulated as RDF triples (subject→predicate→object), which could be arbitrarily long (Figures 3). Cuebee then transforms the triples into SPARQL queries which may be executed in any tool that supports the SPARQL standard.

Cuebee has a suggestion engine that rapidly queries the ontology schema to suggest relevant concepts as the user begins formulating a query. Furthermore, it lists all the relationships that are relevant for a selected concept. Both these features reduce the need for users to be acquainted *a priori* with the ontology and SPARQL query language – a major concern for life science data retrieval systems (Figure 3). Cuebee executes the formulated query in a second query engine, which we refer to as the answer engine, and the results are displayed using different visualization methods such as tables, pie charts or graphs, although we predominantly utilize a tabular format.

While Mendes et al. [28] provide evidence about the improved usability of Cuebee in comparison to the existing lab solutions, several infrastructural modifications and functionalities were added to the basic Cuebee system to make it further usable and useful for biologists. The added functions include support for OWL-based ontologies, interface modifications to support formulating extended queries using Boolean operators, filters, and selection of specific instances and literals. It also facilitates integration of Web services, such as NCBI BLAST, to enrich some of the final results with operations on external data resources. Cuebee also facilitates formulation of complex SPARQL queries such as those that allow grouping of values and applying aggregate functions, such as averaging over the groups, filtering over instances using regular expressions, and support the use of queries that include negation (see query formulation using Cuebee in Results section). In addition, an undo feature has been added to revise queries at any point during the query formulation process and after answers have been generated. The enhanced version is available for use at http://jade.cs.uga.edu.

Support for OWL ontologies was included in Cuebee because OWL-based ontologies tend to be more expressive than those in plain RDF schema, in part due to the allowed use of restrictions in OWL. The OWL ontologies, such as PEO and OPL, are deployed in a popular OWL reasoner called Pellet [27] in order to take advantage of Pellet's inferencing capabilities, which involves using general rules to produce new information. However, a concern in solely relying on Pellet is its lack of scalability in supporting large datasets. In order to support large datasets without sacrificing inferencing capabilities, a scalable RDF storage system called Virtuoso (http://virtuoso.openlinksw.com/dataspace/dav/wiki/Main/) was used as one of our query endpoints. Subsequently, we utilize Pellet that hosts the ontology schemas for our suggestion engine and Virtuoso for hosting and querying the large amounts of data.

In life sciences, there are a large number of bioinformatic tools and data sources available as Web services (for e.g., see BioCatalogue [30]). Cuebee facilitates the use of bioinformatic tools such as NCBI BLAST [31] and TriTrypDB's services, both of which are available as RESTful Web services. If the results of a query contain appropriate types of gene ids or gene sequences, then Cuebee allows the user to trigger an asynchronous invocation of a composition consisting of TriTrypDB's Web services followed by NCBI BLAST. Web services from TriTrypDB are used to obtain the gene and protein sequences given gene ids, which are then sent over to NCBI BLAST. BLAST results generated remotely are retrieved and displayed to the user. A recorded video demonstration of Cuebee with such functionality is available at our Trykipedia website (http://wiki.knoesis.org/index.php/Trykipedia).

### Provenance Capture in SPSE

Provenance information describes the experimental protocol (such as the conditions under which an experiment dataset was produced), type of instruments used for the experiment, and tools used to process or analyze the data. Such provenance information is useful for documenting, reproducing, and tracking protocols over a project and for comparison of data collected using different protocols and in different laboratories [15].

**Figure 4:**
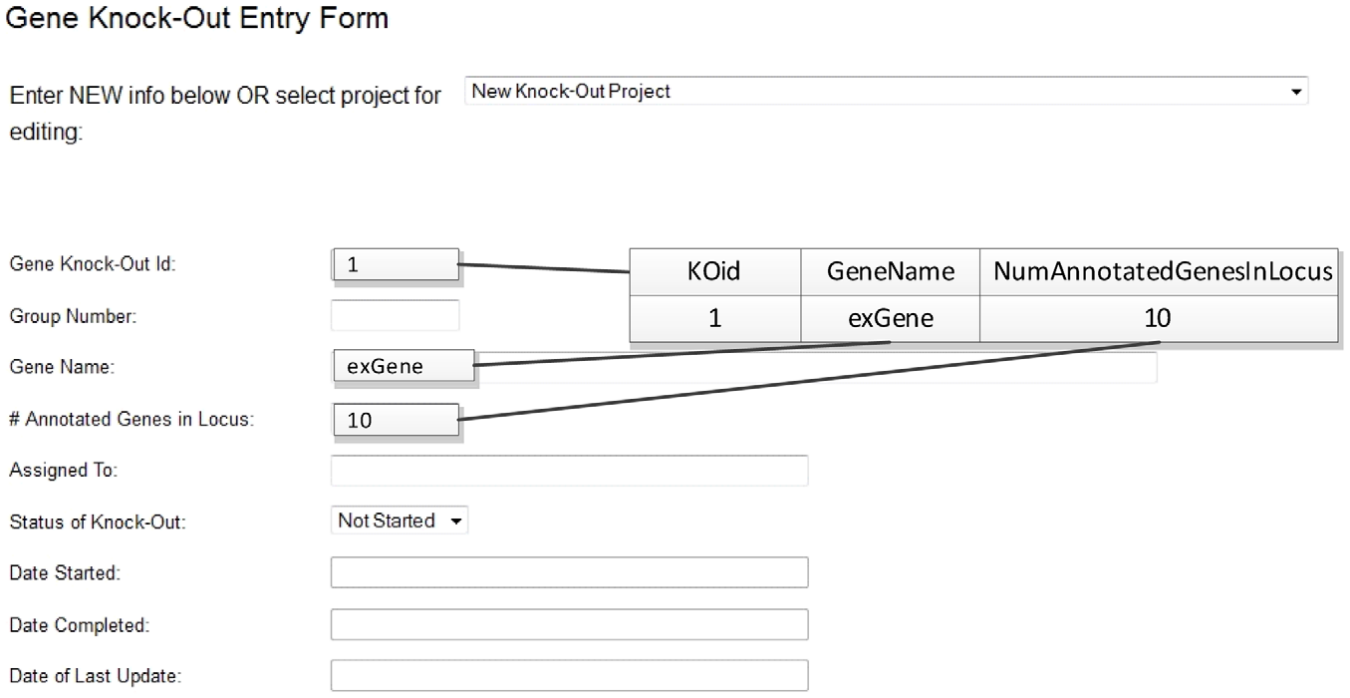
Old Web forms in the lab that stored the experimental provenance data in a conventional relational database.

**Figure 5:**
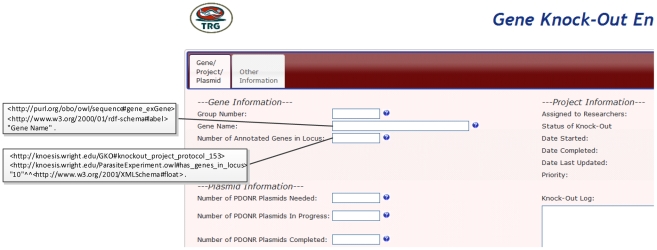
New Web forms that store the data in a RDF subject-predicate-object (i.e., triple) format providing opportunity to relate the data to ontology concepts. Storing the data using these Web forms has no impact on the front-end user experience, but it offers extended querying functionality through the use of ontology concepts. Provenance information added through these Web forms is instantly available for querying.

Experimental data and the associated provenance information were previously collected in the lab using Web-based forms that stored the data in a relational database (Figure 4). This dataset included details of an experiment, such as name of the knock-out target genes, experiment ID, status, responsible personnel, type and number of plasmid used, etc. One approach is to use a batch process to convert the data stored in the relational database to RDF format. However, periodic transformation of data stored in the relational database to RDF is time-consuming and inefficient. Hence we created a set of Web forms based on the PEO schema to directly store the provenance information in RDF format (Figure 5). This allows researchers to query, using Cuebee, the most current version of data available, while avoiding the delay associated with the earlier batch transformation of data to RDF.

## Results

To demonstrate the significance of our SPSE, we set up three complex biological queries that utilize both internal (unpublished/private experimental and provenance) and external (publicly available) data, and execute them using Cuebee. The SPARQL source of all the queries as well as a link that demonstrates the queries in Cuebee are available online at http://wiki.knoesis.org/index.php/Manuscript_Details. Cuebee queries for the first two questions are also displayed below as examples.

*Question 1*: List the genes that are downregulated in the epimastigote stage and exist in a single metabolic pathway.

*Significance*

The answer to this question would help direct efforts to identify genes or pathways that could be targeted for knockout in the epimastigote stage of *T. cruzi* where these genes appear to be expressed at low levels and thus possibly not essential in this stage. The second part of the question addresses the ease of interpreting gene knock-out phenotypes and reduces the odds that essential genes are in the result set. Phenotype results from a knock-out of a gene involved in a single metabolic pathway would be easier to interpret than results from a knock-out of a gene that is involved in multiple pathways. Moreover, genes involved in multiple metabolic pathways are more likely to be essential, and thus not suitable for the production of genetically attenuated vaccine strains, for example.

*Prevailing Approaches*: Multi-stage transcriptome data are available on TriTrypDB so this question may be transformed into a query that can be executed on TriTrypDB. However, it cannot be executed on TriTrypDB as worded, because RNA expression data are presented in the format of stage/stage and not as each stage versus the average expression in all stages. Additionally, identifying genes in a single metabolic pathway only is tedious in TriTrypDB because it would require selecting all genes associated with a metabolic pathway and then post-processing to sort by the number of metabolic pathways associated with each gene in the list.

The gene expression data generated in the lab are stored textually, complicating its retrieval because it is not straightforward to devise a text search that would capture “all genes downregulated in epimastigotes”. Moreover, these lab data are not linked to metabolic pathway information.

*SPSE Approach*: In order to answer this question, we need data from gene expression lab experiments integrated with the pathway information from the KEGG database. When integrating the pathway information from the KEGG, SPSE aligns the “gene” used in the expression data with the “pathway gene” found in the KEGG database using *same (instance)* relationship in PEO. Because of this integration, we may transform the question into a single query using Cuebee and execute it. The query first searches for genes that appear in the epimastigote stage gene expression data and are associated with a single pathway (expressed as a sub-query). These data are then refined in the top-level query to include genes with a microarray log2 ratio that is less than −1.

*Query in Cuebee*

Figure 6

**Figure 6:**
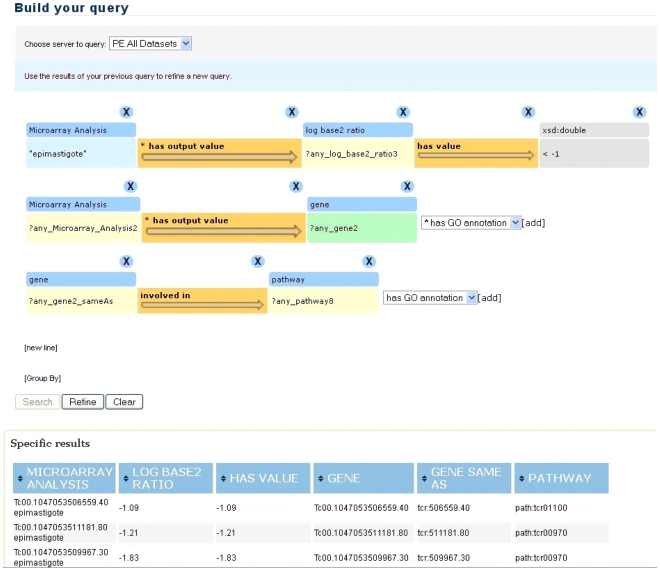
Screenshot of the Cuebee interface after formulation of the query 1, “List the genes that are downregulated in the epimastigote stage and exist in a single metabolic pathway.” Each row contains triples that are required to formulate the query. The query formulation is initiated by first selecting the server (PE All Datasets). After selecting the dataset, users begin to type in the search field and Cuebee provides suggestions matching the first letters typed in a drop down list. In this case “Microarray Analysis” is selected, and the query was limited to microarray analysis data pertaining to only “epimastigote” lifecycle stage of the parasite using filtering function of Cuebee. The triples are then extended as shown to achieve the desired query. The query uses “Group by” function of Cuebee to group all the epimastigote genes associated with a single metabolic pathway and “Refine” function to identify only those genes from the group that are downregulated; i.e, with log2 ratio less than −1. Specific results show a part of the results that include gene information from microarray lab data and pathway information from KEGG where each pathway ID represents specific pathway in KEGG.

*Question 2:* List the summaries of gene knock-out attempts, including both plasmid construction and strain creation, for all gene knock-out targets that are 2-fold upregulated in amastigotes at the transcript level and that have orthologs in *Leishmania* but not in *Trypanosoma brucei*.

*Significance*

This question deals with the routine oversight of high throughput gene knock-out attempts [24] by asking the general question, “What is the status/progress of the experiments involving these genes with these specific characteristics?” There may be multiple gene knock-out targets currently being pursued, which are at different stages in the gene knock-out protocol. In order to look at the current status of all targets in a target group, we need to look at both the plasmid construction and strain creation summaries. Orthologs are genes in different species that share the sequence homology. The orthology of *T. cruzi, T. brucei, and L. major* on TriTrypDB was determined by TRIBE-MCL analysis, which was performed as part of the genome sequencing effort [32]. The rationale for looking at *T. cruzi* genes with orthologs in *Leishmania* and not in *T. brucei* is that these two kinetoplastid parasite groups (*Leishmania* and *T. brucei*) are close phylogenetic relatives of *T. cruzi*, yet, unlike *T. cruzi* and *Leishmania*, *T. brucei* has no intracellular stage in its life cycle. Thus, genes with orthologs in *Leishmania* and *T. cruzi* but not in *T. brucei* might be expected to be important for survival and replication of intracellular parasites. We expect that knocking out such genes will produce parasite strains that are capable of being propagated as epimastigotes but deficient in maintaining infections in mammalian hosts.

*Prevailing Approach*: Answering this question is not possible using existing external databases because the query requires private lab-specific data that are not publicly available. Additionally, as noted for Question 1, the particular presentation of RNA expression data in TriTrypDB complicates this matter. For evaluation purposes, this question was executed in the lab using Boolean searches of lab databases and manual annotation of the orthology data.

*SPSE Approach*: Answering this question requires integration of private tracking data on gene knockout constructs and parasites strains, gene expression data and TriTrypDB data, which we accomplished using PEO as the underlying shared schema. The question also seeks provenance information (i.e., gene knock-out summaries) from the private lab data. The query using Cuebee first searches for the genes for which knock-out (KO) plasmids have been constructed and strains containing these KOs created. Among these, it selects the genes that have at least a 2-fold enhanced expression in amastigotes and have orthologs, which derive only from *Leishmania*.

*Query in Cuebee*

Figure 7

**Figure 7:**
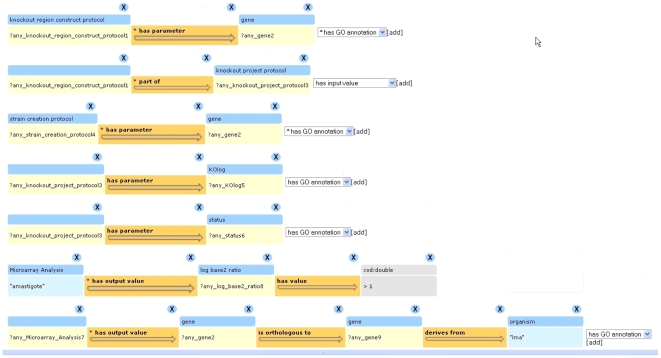
Screenshot of the Cuebee interface after formulation of the query 2, “List the summaries of gene knock-out attempts, including both plasmid construction and strain creation, for all gene knock-out targets that are 2-fold upregulated in amastigotes at the transcript level and that have orthologs in *Leishmania* but not in *Trypanosoma brucei*.” Each row contains triples that are required to formulate the query. The query formulation is initiated by first selecting the server (PE All Datasets). After selecting the dataset users begin to type in the search field and Cuebee provides suggestions matching the first letters typed in a drop down list. In this case “knockout region construct protocol” is selected. The triples are then extended as shown to achieve the desired query. The query uses the “Group by” function of Cuebee to group all the genes that are 2-fold upregulated in amastigote and negation function to identify only those that have orthologs in Leishmania and not in *T. brucei*.

*Question 3*: Provide a summary for all parasite strains created through single or double knock-out of a gene annotated as encoding a protein kinase.

*Significance*

This query combines both private provenance data (strain summaries) and external data (predicted function – protein kinase). The strain summaries are textual information resulting from researchers making specific notes on the status of strains they are in the process of making. The results would be useful for quickly ascertaining the status of all ongoing knock-out projects involving genes encoding protein kinases. Protein kinases in general are considered potentially useful gene knock-out targets, because many protein kinases are well known as master regulators, which affect multiple molecular pathways. Thus, selecting protein kinases that are expressed in *T. cruzi* life-cycle stages that occur in mammalian hosts is expected to produce strains that are attenuated.

*Prevailing Approach*: Similar to Question 2, answering this question is also not possible using existing external databases because the query requires internal data. This query is not possible in TriTrypDB, because the database does not contain provenance data. Furthermore, this query is tedious to perform in the lab using existing infrastructure because it would require first downloading all genes with the term “protein kinase” in their annotation and then manually searching each gene ID in the GKO database, which is separate from the annotation database.

*SPSE Approach*: This question requires the integration of strain creation data along with their provenance information and TriTrypDB. In addition, it uses GO terms to identify the gene of interest. It first searches for the protein kinase genes using integrated TriTrypDB data followed by a search for information on strains developed through knock-out of the genes identified at the earlier stage.

### Evaluation

The results obtained from the above queries were evaluated manually by the parasitologists. The results of Question 1 were compared against the gene knock-out target list that was determined using a mixture of manual data mining strategies, including searching transcriptomic and proteomic expression data for expression in the appropriate life-cycle stages, searching orthology data for conservation among other kinetoplastid organisms, determining gene copy number, and identifying chromosome location to select for genes that are within regions of high-confidence sequence data. We observed overlaps in the results we obtained. Question 1 resulted in 55 genes, of which four were already under consideration for gene knock-out studies in our lab. However, in querying PKB we identified several genes associated with only one metabolic pathway. This could not be done previously with the manual list that was prepared, because of the inability to determine the number of metabolic pathways a particular gene is associated with without manually looking at one gene at a time. Based on the results we obtained, these four genes are now a higher priority for knock-out studies in our lab.

Questions 2 and 3 are routinely used in the lab to learn the knock-out or strain creation status of a given group of genes or how the knock-outs were prepared. The results of both the queries were verified by a combination of using the existing database infrastructure and manually searching lab notebooks.

## Discussion

Parasitic diseases create tremendous social and economic burdens, affecting individuals in every corner of the globe. However, because patients in poor and developing nations are disproportionately affected by parasitic diseases, research into the basic biology and control of parasites is limited in comparison to research into diseases that affect the rich, such as heart disease and cancer. Nonetheless, sufficient genome-scale research into parasite biology has been performed in recent years to make necessary the development of advanced methods for accessing, analyzing, and interpreting large amounts of parasite-related data. Integration of large data sets, such as genome sequence and transcriptomic data, with internal experimental data is currently beyond the capabilities of most biologists. The heterogeneous formats of such databases and the internal experimental data sets limit the use of these resources and make knowledge discovery even more challenging. Therefore, a platform is required that allows parasite researchers to integrate their unpublished experimental data with external resources and be able to query all of this data together. Goble and Stevens [33] analyzed the advantages and disadvantages of current approaches for data integration such as a service-oriented architecture (SOA), link integration, data warehousing, view integration, mashups, and the Semantic Web. Among these approaches, the Semantic Web, with its ultimate goal of creating a unified Web of data that can be queried, emerged as the most feasible approach. This approach has been adopted [11], [12] to create an integrated knowledge base using Semantic Web technologies. Few efforts have been made to provide an integrated platform for parasite researchers that help enhance knowledge discovery in this domain [5], [39]. However, use of these platforms requires researchers to publish their data first, which may not be always plausible. Furthermore, through our experiences we realized that to validate and verify the data, not only raw experimental data but also supporting data (or metadata) are required. For example, to verify microarray data, information about nucleotide sequence present on the array, samples, sample treatments, etc. is also very important [34]. Such metadata describing the history or lineage of a data set, (i.e., provenance) enables human validation of the data quality and verification of the experimental procedures and other parameters that generated those data. Such detailed domain-specific provenance (i.e., semantic provenance) is not supported by existing parasite platforms.

Other semantic Web based systems exist that focus on queries to provide targeted access to data in the life sciences and other contexts. These include query tools such as OpenLink iSPARQL (http://demo.openlinksw.com/isparql/) and NITELIGHT [35], both of which provide graph-based interfaces for query formulation. A user generates a visual graph by adding concepts and connecting them together using relationships. iSPARQL is freely available and Kiefer et al. [36] evaluate it on a single data set. However, to the best of our knowledge, none of these systems evaluated their usefulness on use cases or are in use. Similar to Cuebee, GINSENG [37] offers suggestions to users but from a different perspective. GINSENG relies on a simple question grammar, which is extended using the ontology schema to guide users to directly formulate SPARQL queries. Bernstein et al. [37] briefly evaluated GINSENG on three aspects: usability of the system in a realistic task, its ability to parse large numbers of real-world queries, and its query performance. The experimental results did not compare GINSENG to other systems, and no real-world use of the system has been reported.

Semantics-based approaches also exist that focus more on the data integration aspect than on querying in the life sciences context. GoWeb [38] is a semantic search engine for the life sciences which combines classic keyword-based Web search with text-mining and ontologies to explore result sets and facilitate question answering. Dietze et al. [38] evaluated GoWeb on three benchmarks: BioCreAtIvE 1 (Task 2) [39] in the context of genes and functions, the study by Tang et al. [40] in the context of symptoms and diseases, and the questions from the 2006 TREC Genomics Track [41]. GoWeb provided answers with a recall of 58.1%, 77%, and 78.6% respectively. BioGateway [42] composes several online (such as OBO foundry [43] and GO [5] annotation files) and in-house data sources, and provides a single entry point to query through SPARQL. Cheung et al. [44] introduce semantic Web query federation in the context of neuroscience. Their approach focuses on providing facilities to integrate different data sources and offers either SPARQL or SQL query interfaces to access remote data. However, the usefulness of the system has not been demonstrated in a real-world context. While the above systems operating in the context of life science data are available for public use, we did not find evidence of these systems being used by life science researchers. Furthermore, there is a general lack of explicit comparisons between these approaches and traditional systems. Thus, while Cuebee is not alone in its effort to bring knowledge-driven approaches to the life sciences, we believe that our case study of the system in use is novel.

We developed an SPSE using Semantic Web technologies and provenance primarily *(i)* to address the need for a platform that allows parasite researchers to query their internal data along with external resources while placing minimal computing load on them, and *(ii)* to capture semantic provenance of the experimental data. The current version of SPSE facilitates integrating and querying internal data with external databases, such as TriTrypDB and KEGG using PEO, GO, and PW. SPSE also collects detailed provenance information using a set of Web forms as described previously and supports provenance queries as exemplified by Questions 2 and 3 in the Results and Evaluation section. Furthermore, Cuebee provides a platform to execute complex SPARQL queries visually on RDF data using the ontology schema. This allows biologists to query their data sets with minimal programming skills. Finally, results of the three queries showed that querying PKB provides valuable information on what genes to knock out and thus help find intervention targets in *T. cruzi*, each of which previously involved several steps. PKB can easily be extended to add more parasites or more data sets by extending PEO or aligning PEO with other existing ontologies. We believe that SPSE will enable parasitologists to get more benefit out of the public resources, thereby enhancing parasite research.

### Limitations and Future Work

Based on our extensive discussions with researchers, we identified two important limitations in the SPSE. First, the experimental data that researchers produce in their lab are currently being transformed into RDF programmatically by computer scientists. These experimental data are in various formats, such as flat file or Excel format, or in relational databases. In order to make accessing these data more seamless, the conversion process should be streamlined by either developing a tool or using one of the current tools that convert various formats of experimental data into RDF [45]. This will allow the users of SPSE to reduce their dependence on computer science personnel for integrating new data into the SPSE.

Second, and more importantly, we observed that biologists require some knowledge of the structure of the ontology schema in order to effectively use Cuebee to run complex queries. Although demonstrations of using Cuebee to execute common queries have been provided, this need may require biologists to undergo some training on Cuebee and become well acquainted with the underlying ontologies. This is particularly crucial in the case of PEO, which focuses on the experiment processes and parameters that may not be well known to all users. Our goal, therefore, is to make the interface of Cuebee more natural by providing a preliminary capability to write queries in natural language. This will involve chunking the questions in natural language and relating the chunks to concepts within the PEO and OPL ontologies. We believe that this will enhance the utility of Cuebee and will provide a simpler interface for its biologist users.
